# A personalized home-based exercise training program in children with Marfan and Loeys-Dietz syndromes improves aerobic exercise capacity and health-related quality of life

**DOI:** 10.1186/s13023-026-04234-4

**Published:** 2026-02-04

**Authors:** Thomas Edouard, Fernanda Bajanca, Clara Flumian, Fabrice Marion-Latard, Clément Pradayrol, Aitor Guitarte, Maud Langeois, Philippe Khau Van Kien, Armelle Yart, Françoise Auriol, Eric Garrigue, Yves Dulac

**Affiliations:** 1 Reference Center for Marfan Syndrome and Related Diseases, Children’s Hospital, Toulouse University Hospital, Toulouse, France; 2 RESTORE Research Center, Université de Toulouse, INSERM 1301, CNRS 5070, EFS, ENVT, Toulouse, France; 3 Unit of Pediatric Clinical Research, Children’s Hospital, Toulouse University Hospital, Toulouse, CIC1436 France; 4 Department of Respiratory Function and Sports Medicine, Toulouse University Hospital, Toulouse, France; 5 Medical Genetics Unit, Nîmes University Hospital, Nîmes, France; 6 Medical Genetics Unit, Genève University Hospital, Genève, Switzerland; 7 Department of Endocrinology, Bone Diseases and Genetics, Children’s Hospital, University Hospital of Toulouse, Toulouse, France

**Keywords:** Marfan syndrome, Loeys-Dietz syndrome, Personalized home-based training program, Aerobic exercise capacity, Peak oxygen consumption, Ventilatory anaerobic threshold, Health-related quality of life

## Abstract

**Background:**

Children and adolescents with Marfan (MFS) and Loeys-Dietz (LDS) syndromes report chronic fatigue and reduced physical endurance, which significantly impact their health-related quality of life (HRQoL). We hypothesized that a tailored physical training program could improve these parameters. To test this hypothesis, we conducted an interventional, prospective, single-center clinical trial consisting of a 3-month observation period followed by a 6-month intervention period, during which a personalized home-based training program was implemented. The primary endpoint was the change in ventilatory anaerobic threshold (VAT) assessed during a maximal exercise test. Secondary outcomes were changes in 6-minute walk test (6MWT) distance and HRQoL parameters (assessed before and after intervention using the Pediatric Quality of Life Inventory, PedsQL™) and cardiac tolerance.

**Results:**

A total of 28 children (25 with MFS and 3 with LDS) were enrolled, of whom 19 (68%) completed the study. At baseline, VAT and 6MWT distances were significantly impaired compared to the general population (*p* < 0.001 by one-sample t-test for both parameters), in particular in patient with a systemic score ≥ 7. During the program, there was an overall significant increase in VAT (*p* < 0.001 by ANOVA) and 6MWT distances (*p* = 0.02 by paired t-test). These improvements were accompanied by a significant enhancement in HRQoL parameters in the different dimensions assessed. No changes were observed in maximum heart rate, maximum systolic blood pressure and aortic sinus diameter.

**Conclusions:**

This 6-month personalized home-based exercise training program significantly improved aerobic physical capacity and HRQoL in children with MFS and LDS without affecting aortic sinus diameter. Despite the small number of patients included, which is a common challenge in studies conducted on children with rare diseases, these findings provide promising perspectives for the management of these patients.

**Clinical trial registration:**

URL https://clinicaltrials.gov/; Unique identifier NCT03236571 date of registration 28/07/2017.

**Supplementary Information:**

The online version contains supplementary material available at 10.1186/s13023-026-04234-4.

## Background

Although rare, with an estimated prevalence of 1 in 5,000 to 10,000 live births, Marfan syndrome (MFS; MIM 154700) is one of the most common heritable connective tissue disorders. Manifestations involve multiple organ systems, including the eyes (i.e., severe myopia and lens dislocation), the cardiovascular system (i.e., aortic aneurysm and dissection) and the musculoskeletal system (i.e., tall stature, pectus deformities, scoliosis, flat feet) [1]. This autosomal dominant disorder is caused by a mutation in the gene encoding type 1 fibrillin (*FBN1*), which is a major component of the extracellular matrix. The diagnosis of MFS is based on a combination of clinical features, echocardiographic findings, family history, and genetic testing as defined by the Berlin Consensus Conference in 1986 and subsequently updated in Ghent in 2010 [2]. Recent studies have underscored the pivotal role of dysregulated transforming growth factor-beta (TGF-β) signaling pathway in the pathophysiology of this disease [3]. Notably, abnormalities in genes encoding proteins related to the TGF-β signaling pathway have been linked to phenotypically close syndromes, such as Loeys–Dietz syndrome (LDS). Although MFS and LDS have different genetic bases (while involving the same TGF-β signaling pathway), they share closely overlapping cardiovascular and musculoskeletal manifestations.

Aortic aneurysm and dissection, present in 80% of patients, are the most significant life-threatening complications associated with MFS and LDS. Historically, the diagnosis of MFS was often made late in the course of an aortic dissection in a young adult, which unfortunately resulted in mortality in the majority of cases. Thanks to recent advances in diagnosis and monitoring, as well as the introduction of prophylactic surgical management and beta-blocker treatment, the life expectancy of patients is now similar to that of the general population [4]. Despite these advances, patients still experience significant morbidity, including musculoskeletal impairments, which results in functional limitations and a reduction in overall health-related quality of life (HRQoL) [5–10]. Chronic fatigue and reduced physical endurance are reported by approximately 90% of adults with MFS, which significantly impact their daily activities [8, 9, 11]. Similarly, children and adolescents with either MFS or LDS report increased fatigue and limited participation in physical activities and daily life, which are associated with a reduction in quality of life compared with their healthy peers [12, 13]. In a recent study on children with MFS and LDS, we found a strong and independent correlation between HRQoL and aerobic physical capacity, as assessed by peak oxygen consumption (VO_2_max) and ventilatory anaerobic threshold (VAT) during a maximal exercise test [14].

It has been demonstrated that physical activity and training can be an effective approach for improving muscle strength and functional capacity, as well as reducing fatigue and improving quality of life in patients with chronic diseases, including cardiovascular diseases [15–17]. However, specific exercise training programs for MFS and related disorders are currently limited and primarily designed for adult patients [18–20]. In children, one study has evaluated a physical activity program aiming at achieving 10,000 steps per day in children with MFS, with promising results regarding the evolution of aortic diameters [21]. Similarly, a pilot study in children with heritable connective tissue disorders, including MFS and LDS, showed that a 12-week physical training program was feasible and safe, with promising improvements in physical fitness and symptom burden [22]. Additionally, findings from animal studies also suggest that regular endurance exercise may be beneficial [23, 24].

We hypothesized that a 6-month personalized home-based training program could improve aerobic exercise capacity and HRQoL in children and adolescents with MFS and LDS. To test this hypothesis, we conducted an interventional, prospective, single-center clinical trial. The primary objective was to evaluate the effect of the training program on physical endurance capacity, and the secondary objectives were to assess its effects on quality of life and cardiovascular parameters.

## Methods

### Study design

This study was an interventional, prospective, single-center and single-arm clinical trial conducted at a national pediatric tertiary care reference center for MFS and related conditions. The study comprised two periods: (1) a 3-month observation period followed by (2) a 6-month intervention period, during which a personalized home-based training program was implemented.

During the initial observation period, aerobic exercise capacity was assessed by a maximal exercise test performed three months before (M-3) and immediately before (M0) the intervention. The data obtained during this observation period were used to verify the reproducibility of our assessments and to assess the spontaneous evolution of the parameters over a 3-month period. During the intervention period, changes in the various parameters (in particular aerobic exercise capacity) were assessed at three months (M3) and six months (M6) after the initiation of the program. HRQoL was evaluated at the beginning and end of study (M0 and M6).

The primary endpoint of this study was the change in aerobic exercise capacity assessed by the ventilatory anaerobic threshold (VAT) over the 6-month personalized home-based training program. Secondary endpoints included HRQoL parameters (assessed using the Pediatric Quality of Life Inventory, PedsQL™), exercise capacity (measured by the distance covered during the 6-minute walk test), and cardiac tolerance (evaluated by changes in aortic sinus diameter, heart rate and blood pressure).

### Study population

Inclusion criteria were children or adolescents (aged 7 to 18 years) of either sex with a molecularly confirmed diagnosis of MFS or LDS, ability to exercise, written informed consent signed by at least one of the two legal guardians, and health insurance. Exclusion criteria included severe aortic dilatation (aortic diameter > 45 mm), left ventricular failure (left ventricular ejection fraction < 45%), severe mitral regurgitation (≥ grade 3), participation in another study, and pregnancy. In addition, study discontinuation was required in the event of adverse events or intercurrent illness that prevented compliance with the protocol for the safety and well-being of the patient.

### Clinical, echocardiographic and genetic features of the study population

Personal history, as well as clinical data (including ocular, cardiovascular and skeletal manifestations), were obtained by retrospective chart review. Anthropometric patient characteristics were collected (height and weight with the calculation of body mass index [BMI]). The ‘systemic score’ was calculated in accordance with the 2010 revised Ghent nosology [2]. In addition to aortic disease and *ectopia lentis*, this score includes all other MFS cardiovascular (i.e. mitral valve prolapse) and ocular (i.e. severe myopia) manifestations, as well as findings in other organ systems, such as the skeleton, dura, skin and lungs (i.e. disproportionately tall stature, arachnodactyly, pectus deformity, scoliosis, pes planus, acetabular protrusion, pneumothorax, dural ectasia, skin striae, and facial features). A systemic score ≥ 7 was considered a major diagnosis criterion for MFS.

Echocardiographic measurements (outflow tract diameter, aortic sinuses, sinotubular junction, and tubular ascending aorta) were also collected and converted to z-scores [25]. Aortic dilatation was defined as aortic root z-score > 2.

Molecular analyses were performed in a single reference laboratory, using an NGS panel comprising 35 genes involved in MFS and LDS, as previously described [14].

### Health-related quality of life assessment

HRQoL was assessed using the Pediatric Quality of Life Inventory (PedsQL™) generic questionnaire as previously reported [26]. Different age-adjusted PedsQL™ versions are available, with self-reported questionnaires to be completed by children aged above 5 years and proxy-reported questionnaires to be completed by their parents. Four versions of the PedsQL™ self and proxy questionnaires were used in this study (5–7, 8–12, 13–17 and 18–25 years old). The 23 items in the PedsQL™ include four multidimensional scales: physical (8 items), emotional (5 items), social (5 items) and school (5 items) functioning. Each item uses a 5-point Likert scale from 0 (never) to 4 (almost always). Items are reverse scored and linearly transformed to a 0-to-100 scale, with higher scores indicating a better HRQoL. Two summary scores can be calculated: the psychosocial health summary score and the physical health summary score.

### Six-minute walk test

The six-minute walk test (6MWT), which corresponds to the distance covered during the six-minute walk test, was used to quantify the functional exercise capacity of our patients. The measurements were converted to z-scores in accordance with the published reference values [27].

### Maximal exercise test

Patients performed a maximal exercise test on a cycle ergometer to assess their aerobic physical capacity. A single pediatric cycle ergometer protocol was used to obtain a homogeneous incremental overall duration between 8 and 12 min, as previously described [14]. The exercise test was considered maximal when the following criteria were reached: respiratory exchange ratio (RER = carbon dioxide production [VCO_2_] / oxygen consumption [VO_2_]) ≥ 1.05, the limit of the child’s tolerance despite verbal encouragement, and inability to provide a minimum pedaling frequency of 60 per minute despite verbal encouragement. The maximum heart rate > 85% of the maximum age-predicted heart rate was not used as a criterion for maximal effort in this study, as most MFS patients are on beta-blockers. The values of peak oxygen consumption (VO_2_max) and ventilatory anaerobic threshold (VAT) were collected. VO_2_ max and VAT were expressed in raw values (mL/kg/min) and normalized as a percentage of predicted VO_2_ max according to the height-based pediatric equations from Cooper et al. [28]. A VO_2_max < 80% of predicted values indicated impaired aerobic physical capacity. A VAT < 55% of predicted VO_2_max values supported a finding of physical deconditioning.

### Personalized home-based training program

The training program comprised two 40-minute sessions per week, delivered over a 6-month period (from M0 to M6) on a cycle ergometer at home. Each session included a 5-minute warm-up, six 5-minute sequences and a 5-minute cool-down. Each 5-minute sequence alternated successively four minutes of pedaling at a load corresponding to the first ventilatory threshold (corresponding to the VAT), designated as “Base”, and one minute of pedaling at a load corresponding to the second ventilatory threshold, designated as “Peak”. The first and the second ventilatory thresholds were determined during the initial maximal exercise test. If the second ventilatory threshold was not reached by the subject during the initial testing, the “Peak” was defined as the load corresponding to 80% of the theoretical maximum power. This personalized training program was initiated during a day hospital stay and subsequently conducted independently at the patient’s home with telephone coaching provided by an adapted physical activity (APA) educator. To guarantee the efficacy and safety of the at-home sessions, the APA educator conducted follow-up calls with the patient after each session for the first three weeks, then once a week. Heart rate was monitored during the training session and if it dropped more than 5–10 beats per minute for a given power level, the load was increased by 10% in order to maintain the target heart rate.

The training program on the cycle ergometer was completed by a muscle-strengthening program carried out using elastic bands with handles. It consisted of one session per week of two sets of 10 repetitions for the following muscle groups: spinal chains (large dorsal, paravertebral, abdominal), deep chains (iliopsoas), quadriceps, hamstrings, biceps, triceps.

### Statistical analysis

Statistical analyses were performed using R software version 4.4.2.

For descriptive analyses, numerical variables were summarized as medians with their first and third quartiles (Q1; Q3), and categorical variables as frequencies and percentages for each category.

For comparisons of numerical variables against a reference value, one-sample t-tests or Wilcoxon signed-rank tests were performed, depending on the data distribution.

For comparisons of numerical variables (i.e., baseline exercise capacity parameters) and categorical variables (i.e., impaired VO_2_max and VAT) between groups (e.g., with or without *ectopia lentis* or scoliosis, with systemic score < 7 vs. ≥7, highest vs. lowest responders), Wilcoxon rank-sum test and Fisher’s exact test were performed, respectively.

For comparative analyses involving variables measured at four different time points, a linear mixed model with random intercept and slope was used to account for the matching of repeated measurements. After verifying the model’s fit and quality, an ANOVA based on the model was conducted to assess whether there was an overall difference between time points. Post-hoc tests with Tukey’s procedure were applied for pairwise comparisons.

For paired data (pre- vs. post-program), paired t-tests or Wilcoxon signed-rank tests were used, depending on the distribution of the differences.

Finally, the initial characteristics of patients included in the analysis were compared to those of the 9 patients excluded. Fisher’s exact test was used for categorical variables, and Wilcoxon rank-sum test for numerical variables.

### Patient exclusion from the analysis

A total of nine patients were excluded from the final analysis due to incomplete data. The majority of the missing data resulted from patients and their families deciding to withdraw from the program before its completion. Specifically, four patients dropped out at the end of the observation period (M0), and an additional four withdrew at M3. In one case, a missed visit during COVID-19 confinement prevented the collection of VO_2_ data at M3 and largely affected the implementation of the program, leading to exclusion of the patient’s data. These exclusions were necessary to ensure the integrity and accuracy of the study’s findings.

## Results

### Baseline characteristics of the study population

The main baseline characteristics of the study population are presented in Table 1 and individual patient descriptive data is presented in Supplemental Table 1.

**Table 1:** Baseline characteristics of the children enrolled in the study

Number of patients	All children enrolled in the study	Children who completed the study	Children who stopped the study	*P*-value ^*^
	28	19	9	
**Marfan syndrome / Loeys-Dietz syndrome**	25 (89.3%) / 3 (10.7%)	17 (89.5%) / 2 (10.5%)	8 (88.9%) / 1 (11.1%)	1.0
**Female / male**	11 (39.3%) / 17 (60.7%)	6 (32%) / 13 (68%)	5 (55.6%) / 4 (44.4%)	0.4
**Age** (years)	12.5 (10.3; 15.6)	11.3 (8.3; 17.1)	13.4 (11.1; 14.4)	0.5
**Echocardiography**				
Aortic sinus diameter (z-score) ^†^	2.5 (1.4; 3.3)	2.5 (1.0; 3.2)	3.0 (2.2; 3.6)	0.2
Aortic root dilatation (z-score ≥ 2) ^†^	19 (70.4%)	11 (61.1%)	8 (88.9%)	0.2
**Maximal exercise test**				
Maximum heart rate (beats/min)	164 (149; 180)	164 (146; 184)	164 (157; 173)	0.8
Percent-predicted maximum heart rate (%)	81.8 (73.6; 89.3)	80.8 (71.0; 90.8)	82.8 (81.2; 86.1)	0.5
Respiratory exchange ratio	1.2 (1.2; 1.2)	1.2 (1.2; 1.2)	1.2 (1.2; 1.3)	0.4
VO_2_max (mL/Kg/min)	31.6 (28.6; 38.3)	31.5 (28.6; 42.0)	31.7 (28.6; 36.0)	> 0.9
Percent-predicted VO_2_max (%)	64.2 (52.8; 72.9)	63.1 (45.6; 73.0)	65.3 (54.9; 71.6)	0.7
Impaired VO_2_ max (< 80% of predicted VO_2_max)	25 (89.3%)	16 (84.2%)	9 (100.0%)	0.5
VAT (mL/kg/min)	17.0 (15.7; 21.3)	16.8 (15.8; 22.0)	17.1 (15.6; 19.4)	> 0.9
Percent-predicted VAT (%)	33.8 (29.0; 39.2)	32.5 (29.0; 38.2)	38.6 (32.2; 40.0)	0.4
Impaired VAT (< 55% of predicted VO_2_max)	27 (96.4%)	19 (100.0%)	8 (88.9%)	0.3
**Six-minute walk test**				
Distance covered (meter)	588 (558; 622)	588 (551; 625)	588 (580; 598)	0.8
Distance covered (z-score)	-0.7 (-1.5; -0.4)	-0.8 (-2.9; 0.4)	-0.7 (-0.8; -0.5)	0.5

Values were expressed as number of patients (%) or median (Q1; Q3). VAT, ventilatory anaerobic threshold; VO_2_max, peak oxygen consumption

^*^Comparison of children who completed and stopped the study: P-values were calculated using a Fisher’s exact test for categorical variables, and Wilcoxon rank-sum test for numerical variables. ^†^Information available in only 27 patients (one patient had undergone prophylactic aortic surgery and completed the study)

#### Demographics and anthropometrics

A total of 28 patients (11 females [39.3%], 17 males [60.7%]) with a median (Q1; Q3) age of 12.5 (10.3; 15.6) years, 25 with MFS and 3 with LDS, were included in this study. The median height z-score was 3.0 (2.2; 4.0), indicating a significantly taller stature relative to age-matched peers. Conversely, the median BMI z-score was − 1.4 (-2.5; 0.7), reflecting a leaner body composition.

#### Systemic and cardiac features

Half of the patients (14 patients, 50%) had a systemic score ≥ 7, which aligns with a more severe multisystemic presentation. A history of pneumothorax was reported in three patients with MFS between the age of 13 and 14 years. Ocular involvement was common, with 13 patients (46%) exhibiting *ectopia lentis*, and 8 of them (29%) subsequently requiring lens surgery.

Regarding cardiac therapy, the majority of patients (26 patients, 93%) received prophylactic treatment, with all cases involving the administration of a beta-blocker. Structural abnormalities of the mitral valve, without significant mitral regurgitation > grade 2, were identified in 16 of the patients (57%), while aortic root dilatation was observed in nearly two-thirds of the patients (19 patients, 68%), with a median aortic sinus diameter z-score of 2.5 (1.4; 3.3). One patient had undergone prophylactic aortic surgery prior to entering the training program, and there were no cases of aortic dissection.

#### Molecular diagnosis

The majority of patients (25 patients, 89%) carried a pathogenic variant in the *FBN1* gene consistent with the diagnosis of MFS. Of these, 9 (36%) exhibited premature termination codon variants and 16 (64%) having in-frame variants. Among the in-frame variants of *FBN1*, 8 (50%) were cysteine loss variants, 2 (12.5%) cysteine gain variants, 4 (25%) non-cysteine gain variants and 2 (12.5%) in-frame deletion or duplication variants. In 16 of the 25 patients (64%) with *FBN1* variant, the variant was inherited from a heterozygous parent. The remaining pathogenic variants (3 patients, 11%) affected genes involved in TGFß signaling pathway (1 patient with a *de novo* variant in *TGFßR1* gene and 2 inherited variants in *SMAD3* gene), consistent with the diagnosis of LDS.

#### Baseline exercise capacity

The aerobic exercise capacity of the 28 children was significantly impaired at baseline compared with normative pediatric values. The median (Q1; Q3) 6-minute walk test (6MWT) distance was 588 (558; 622) meters, corresponding to a z-score of -0.7 (-1.5; -0.4) (*p* < 0.001 compared with the general population by one-sample t-test). During maximal exercise test, the median VO_2_max was 31.6 (28.6; 38.3) mL/kg/min, representing 64.2 (52.8; 72.9) % of the predicted values (*p* < 0.001 by one-sample t-test). The median ventilatory anaerobic threshold (VAT) was 17.0 (15.7; 21.3) mL/kg/min, representing 33.8 (29.0; 39.2) % of the predicted values (*p* < 0.001 by one-sample t-test). Impaired VO_2_max or VAT was observed in 89% and 96% of patients, respectively (Table 1).

Patients with a systemic score ≥ 7 (*n* = 14), indicating more severe skeletal involvement, exhibited a significantly lower percent-predicted VAT at baseline compared with those with a systemic score < 7 (*n* = 14) (*p* = 0.021) (Supplemental Table 2). In contrast, the distance covered during the 6MWT (expressed in z-score) and the percent-predicted VO₂max did not differ between these 2 groups (*p* = 0.4 and 0.069, respectively). No significant differences in baseline exercise capacity (6MWT, VO₂max, and VAT) were observed between patients with or without scoliosis (18 vs. 10) or with or without *ectopia lentis* (13 vs. 15). Given that the majority of patients (93%) were receiving beta-blocker therapy, the effect of this treatment on baseline exercise capacity could not be assessed. Similarly, it was not possible to assess the potential impact of a history of pneumothorax due to the small number of affected patients (*n* = 3).

### Evolution during the personalized home-based training program

#### Adherence to the personalized home-based training program

Of the 28 children who were initially enrolled in the study, only 19 (68%) completed the 6-month training program and were included in the analysis of the effect and tolerability of the program. The decision to withdraw 9 children from the study was taken due to the difficulty in maintaining consistent and substantial involvement of the children and their families. No discontinuation was related to an adverse event. There were no significant differences in the baseline characteristics between the children who completed the study and those who withdrew (Table 1, Supplemental Table 3).

#### Changes in exercise capacity

**Fig. 1:**
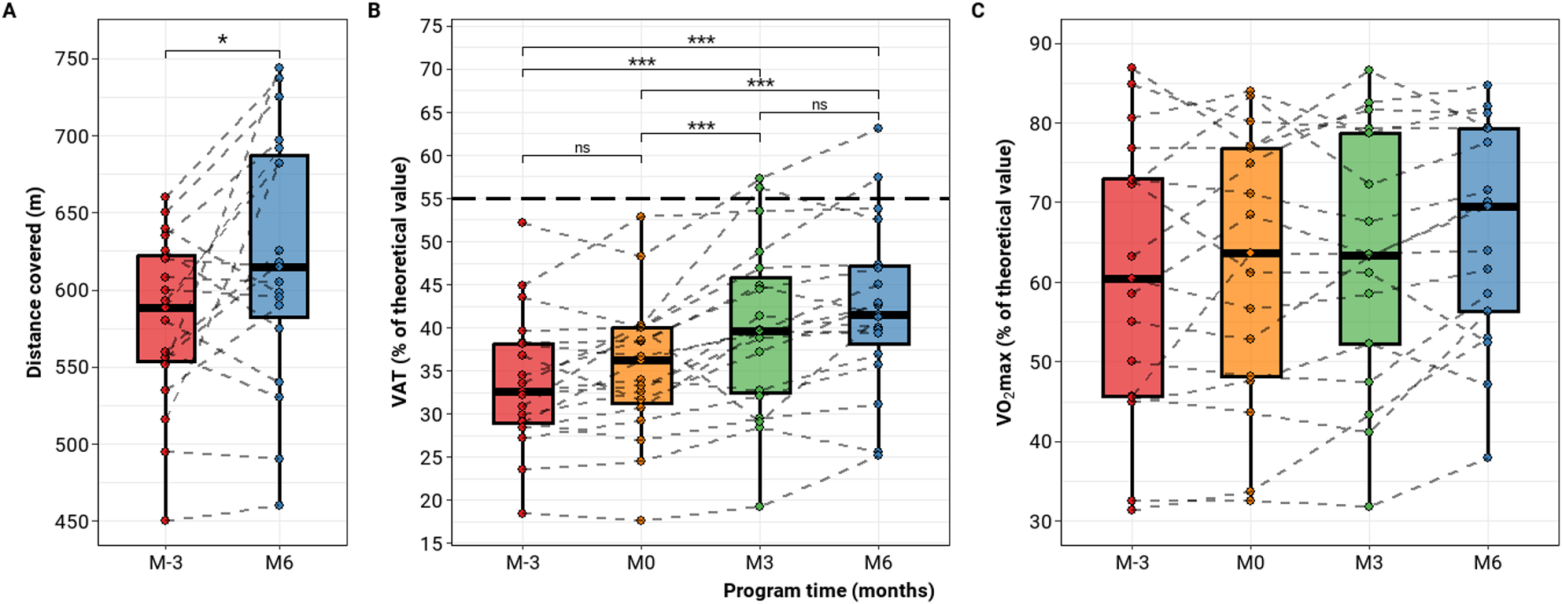
Evolution during the training program of (**A**) the distance covered during the 6-minute walk test, (**B**) the ventilatory anaerobic threshold (VAT), and (**C**) the peak oxygen consumption (VO2max) during a maximal exercise test

During the program, the 19 children who completed the 6-month training exhibited significant improvements in endurance capacity, as measured by the 6MWT and the exercise test (Fig. 1). The mean difference (95% CI) in the 6MWT distance between baseline (M-3) and the end of the program (M6) was 41 m (8; 74), which was statistically significant (*p* = 0.02, paired t-test) (Fig. 1A).

VAT also increased significantly during the training program in all patients (*p* < 0.001, ANOVA). Post-hoc comparisons using the Tukey procedure showed significant differences between all time points, except during the observation phase (M-3 to M0; *p* = 0.46) and the final three months of the program (M3 to M6; *p* = 0.079) (Fig. 1B**).** Despite this increase, only two patients (10%) reached normal VAT levels during the program (≥ 55% of predicted VAT). The median (Q1; Q3) percentage change in percent-predicted VAT between M-3 and M6 was 23.7% (11.7; 37.2). Comparison of baseline characteristics (age, sex, systemic score, presence of scoliosis or *ectopia lentis*, and baseline exercise capacity parameters) between the highest responders (≥ 10% change in percent-predicted VAT; *n* = 8) and the lowest responders (*n* = 11) revealed no significant differences.

In contrast, VO₂max values did not change significantly over the program (*p* = 0.15, ANOVA) (Fig. 1C), suggesting that while endurance capacity improved, maximal aerobic capacity remained stable over the study period.

#### Impact on health-related quality of life (HRQoL)

**Fig. 2:**
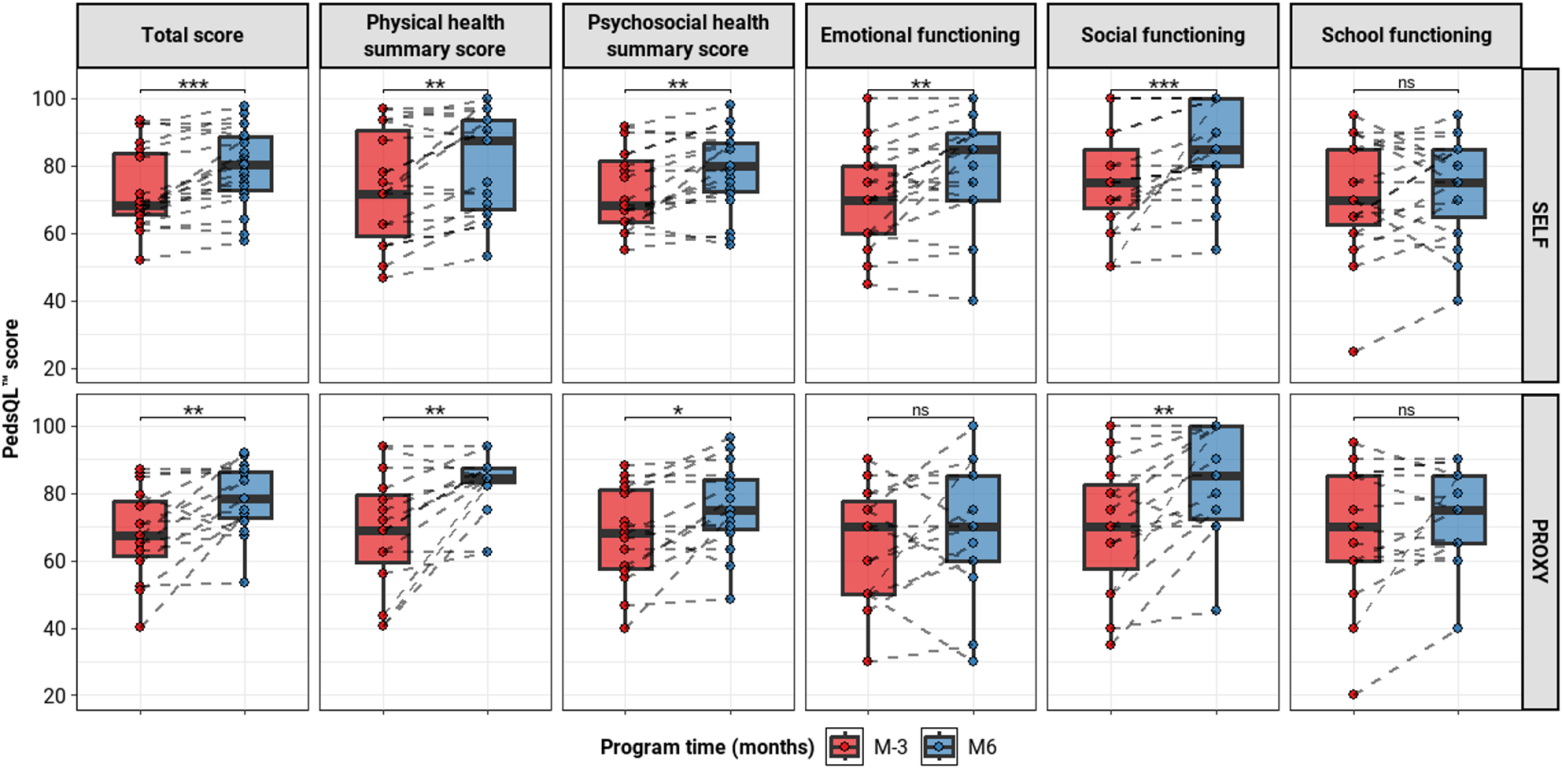
Self and proxy-reported PedsQL™ scores before and after the training program

Interestingly, this improvements in physical endurance capacity were accompanied by a significant improvement in HRQoL across most accessed dimensions, except for the school functioning dimension (Table 2; Fig. 2). The most notable improvements were observed in physical health summary score and social functioning domains, reflecting better overall well-being and engagement in daily activities.

**Table 2:** Self and proxy-reported PedsQL™ scores before and after the training program

	Self-reports			Proxy-reports		
	Before	After	*P*-value ^1^	Before	After	*P*-value ^*^
Total score	68.5 (65.8; 83.7)	80.4 (72.8; 88.6)	**< 0.001**	66.3 (57.9; 76.9)	78.3 (72.8; 86.4)	**0.005**
Physical health summary score	71.9 (59.4; 90.6)	87.5 (67.2; 93.8)	**0.002**	68.8 (53.1; 78.9)	84.4 (83.3; 87.5)	**0.003**
Psychosocial health summary score	68.3 (63.3; 81.7)	80.0 (72.5; 86.7)	**0.002**	67.5 (57.9; 80.4)	75.0 (69.2; 84.2)	**0.02**
Emotional functioning	70.0 (60.0; 80.0)	85.0 (70.0; 90.0)	**0.006**	65.0 (50.0; 76.2)	70.0 (60.0; 85.0)	0.31
Social functioning	75.0 (67.5; 85.0)	85.0 (80.0; 100.0)	**< 0.001**	70.0 (53.8; 81.2)	85.0 (72.5; 100.0)	**0.004**
School functioning	70.0 (62.5; 85.0)	75.0 (65.0; 85.0)	0.37	70.0 (60.0; 85.0)	75.0 (65.0; 85.0)	0.19

Values were expressed as median (Q1; Q3)

^*^Comparison of the HRQoL parameters before and after the training program: P-values were calculated using a paired t-test when the distribution of differences follows a normal distribution, otherwise paired Wilcoxon rank test

#### Safety and tolerability of the program

**Fig. 3:**
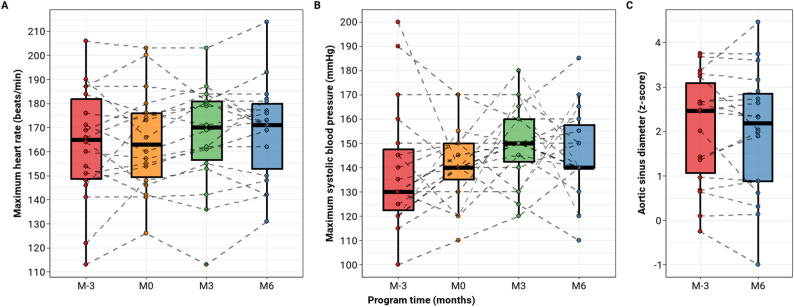
Evolution during the training program of (**A**) maximum heart rate, (**B**) maximum systolic blood pressure, and (**C**) aortic sinus diameter

No adverse effects were reported during the study, including cardiac, ocular, or musculoskeletal complications. In particular, key safety parameters such as the maximum heart rate (*p* = 0.23 by ANOVA), maximum systolic blood pressure (*p* = 0.20 by ANOVA) and aortic sinus diameter (*p* = 0.60 by paired t-test), remained stable (Fig. 3). These findings support the safety of personalized home-based training in this population.

## Discussion

This study represents the first evaluation of the efficacy and tolerability of a personalized home-based exercise training program in children and adolescents with MFS or LDS. The findings demonstrate that this training program significantly improved aerobic capacity and HRQoL within only six months, with no adverse effects observed, particularly on aortic sinus diameter.

At baseline, participants exhibited significantly reduced 6MWT distances, VAT and VO_2_max values compared to the general population. 80% of children demonstrated impaired aerobic exercise capacity (defined as VO_2_max < 80% of predicted values) and all were physically deconditioned (defined as VAT < 55% of predicted VO_2_max values). These findings are consistent with data published in adult patients with MFS, who reported a 25% reduction in peak oxygen uptake compared with healthy individuals [20]. Similarly, a reduction in physical capacity, as assessed by maximal exercise test and Fit Kids Treadmill test, has been reported in children with MFS or LDS with a significant correlation between physical capacity and HRQoL parameters [14] and fatigue level [12]. Impaired physical endurance is likely to be multifactorial, with primary muscle defect being a significant contributor. Indeed, several studies have reported a reduction in muscle mass and strength in adults with MFS [29–31]. This deficit in muscle mass is already present in young children and worsens in adolescents and young adults [32]. Histological analyses of skeletal muscle from MFS patients, but also from animal models (*Fbn1*-deficient mice), have shown a reduction in the number and size of muscle fibers associated with a fragmented appearance of these fibers [31, 33]. The TGF-β signaling pathway has been implicated in the regulation of muscle growth, and its excessive activation is linked to muscle atrophy and impaired muscle regeneration [34]. It has been reported that interventions that reduce TGF-β activity, such as anti-TGF-β antibodies or losartan, restore normal muscle architecture and regenerative capacity in *Fbn1*-deficient mice [33]. This suggests that primitive muscle defect in MFS is closely tied to dysregulated TGF-β signaling. Furthermore, the severity of the various ocular, cardiac and musculoskeletal conditions, combined with the recommended physical activity restrictions to minimize the risk of aortic dilatation progression and dissection [1], may exacerbate this underlying muscle deficit. Notably, greater skeletal involvement, as reflected by higher systemic scores, was associated with a more pronounced decline in endurance capacity in our cohort at baseline.

Although numerous studies have reported on the benefits of cardiopulmonary exercise training in the general population and in patients with various cardiovascular diseases [15–17], very few studies have focused on patients with MFS [18–21]. A 6-month physical activity intervention to achieve 10,000 steps per day resulted in a significantly lower rate of aortic root diameter progression in 24 children with MFS [21]. In 18 adult patients with MFS, a first observational study reported the efficacy and safety of a 3-week inpatient low-intensity exercise program [19]. During this program, patients demonstrated significant improvements in physical fitness, as measured by maximum power on a cycle ergometer and maximum Nordic walking distance. Additionally, patients reported positive changes in mental health, fatigue, nociception and vitality. Although aortic diameters were not measured, no medical issues or adverse events were noted. More recently, a randomized and controlled study was conducted to assess the impact of a 3-month online personalized home-based training program on HRQoL and cardiovascular and muscular capacity in 52 adult patients with MFS, of whom 38 patients were in the training group and 14 in the control group [20]. The training program significantly improved the HRQoL, exercise capacity, muscle strength, and arterial compliance while no changes were observed in aortic root diameter. Interestingly, a beneficial effect of exercise on the aortic root dilation and left ventricle function has also been suggested in a mouse model of MFS [23]. In this model, a 5-month moderate-intensity training slowed the progression of aortic root dilatation and improved left ventricle hypertrophy in exercised mice compared with their sedentary counterparts. This finding aligns with a case report demonstrating that a 4-year physical therapy program successfully reversed left ventricle dilatation and hypertrophy in a patient with MFS [18]. It is important to note that the beneficial effects of physical activity have been reported with moderate-intensity training. However, higher-intensity exercise may potentially yield adverse effects. In the mouse model of MFS, exercise at intensities between 55% and 65% of VO_2_max was shown to have a protective effect, whereas higher intensity exercise (75 to 85% of VO_2_max) negatively impacted the elastic fiber structure and aortic wall elasticity [24]. Similarly, our study is the first to demonstrate the beneficial effect of physical exercise on endurance capacity and HR-QoL in children and adolescents with MFS. The program was well tolerated from a cardiovascular perspective; however, no evidence was found to suggest a beneficial impact on aortic diameter progression. These findings are in line with the recent American Heart Association guidelines on pediatric aortopathy, which advocate for the promotion of age-appropriate, safe physical activity from early childhood to support a healthy cardiovascular lifestyle, as well as social development through community engagement and teamwork [35].

One of the limitations of this study was the relatively small number of patients included, which is a common challenge in studies conducted on children with rare diseases. However, despite the relatively small number of children and the short duration of the program, significant differences were found, underlining the effectiveness of this approach. Another limitation is the high number of participants who discontinued the study (29% dropout rate), which was also observed in the study by Jouini et al., with an overall dropout rate of 25% (17% in the group following the program and 42% in the control group) [20]. In our study, the decision by the children and their families to cease participation in the program was influenced by the restrictive and somewhat uninspiring nature of the training program on cycle ergometer. There were no instances where discontinuation was related to an adverse event. We are therefore seeking to evaluate the impact of an adapted physical activity (APA) program that is tailored to the child’s daily routine and interests. By integrating physical activity into the routine of children and young adults, we aim to create lifelong habits that will improve physical endurance and quality of life for these patients.

Due to the low prevalence of the disease, we did not conduct a randomized controlled trial, which would have been the preferred methodological approach. However, we implemented a 3-month observation period for each patient, allowing us to verify the reproducibility of our assessments and assess the spontaneous evolution of parameters over three months.

The resources required to set up this personalized program were significant, including the equipment loan (heart rate monitor watch and cycle ergometer) and regular telephone follow-up by an APA educator to adapt the program and ensure there were no adverse effects. This could make it difficult to extend the program to a wider population. A detailed medico-economic analysis of the program would be beneficial.

## Conclusion

This study highlights the importance of implementing tailored physical training programs for individuals with MFS or LDS starting in childhood. These non-pharmaceutical therapeutic interventions hold significant potential to enhance physical fitness and quality of life for these patients, with a possible positive impact on the progression of aortic root dilation. Moreover, by integrating physical activity into the daily routines of children and young adults, we aim to establish lifelong habits that can positively influence the progression and overall experience of the disease for these patients.

## Supplementary Information

Below is the link to the electronic supplementary material.

Supplementary Material 1

## Data Availability

Any data supporting the findings of this study not provided as main or supplementary tables are available from the corresponding author on reasonable request.
