# Supplementary material for: A personalized home-based exercise training program in children with Marfan and Loeys-Dietz syndromes improves aerobic exercise capacity and health-related quality of life
Source: Orphanet J Rare Dis. 2026 Feb 4;21:86. doi: 10.1186/s13023-026-04234-4 (PMC12958747; doi:10.1186/s13023-026-04234-4)
Supplement: Supplementary file 1 — Supplementary Material 1 [file 13023_2026_4234_MOESM1_ESM.docx]

**Supplemental Table 1.** Individual patient baseline characteristics

| **ID** | **Age** | **Sex** | **Gene** | **Variant type** | **Ectopia lentis** | **Ectopia lentis surgery** | **βblocker** | **Aortic sinus diameter (zs)** | **Height (SDS)** | **BMI (zs)** | **Systemic score** | **Scoliosis** | **Scoliosis surgery** | **Pneumo**  **thorax** |
| --- | --- | --- | --- | --- | --- | --- | --- | --- | --- | --- | --- | --- | --- | --- |
| **1** | 11 | M | *FBN1* | Missense Cys- | yes | no | yes | 2.7 | 4.5 | 0.9 | 8 | no | no | no |
| **2** | 14 | F | *FBN1* | Missense Cys- | yes | no | yes | 1.0 | 2.5 | -2.7 | 4 | yes | no | no |
| **3** | 11 | M | *FBN1* | Missense Cys- | no | - | yes | 2.7 | 0.6 | -2.8 | 7 | yes | no | no |
| **4** | 17 | F | *FBN1* | Missense Cys- | yes | yes | yes | * | 4.0 | -0.2 | 9 | yes | no | yes |
| **5** | 19 | F | *FBN1* | PTC | yes | yes | yes | 4.4 | 2.5 | -1.4 | 12 | no | no | yes |
| **6** | 15 | M | *FBN1* | Missense non-Cys | no | - | yes | 3.4 | 2.7 | -0.2 | 10 | yes | no | no |
| **7** | 12 | M | *FBN1* | PTC | no | - | yes | 2.2 | 3.1 | -3.5 | 6 | yes | no | no |
| **8** | 13 | M | *FBN1* | Missense Cys+ | yes | no | yes | 2.4 | 2.3 | 0.9 | 8 | no | no | no |
| **9** | 13 | F | *FBN1* | PTC | yes | yes | yes | 2.0 | 3.3 | -0.1 | 5 | yes | no | no |
| **10** | 11 | M | *FBN1* | PTC | yes | yes | yes | 1.0 | 4.0 | 0.7 | 0 | no | no | no |
| **11** | 8 | F | *FBN1* | PTC | yes | yes | yes | 3.2 | 2.0 | -1.7 | 2 | yes | no | no |
| **12** | 14 | F | *FBN1* | PTC | no | - | yes | 2.0 | 1.9 | 0.9 | 10 | yes | no | no |
| **13** | 13 | F | *FBN1* | In frame del/dup | no | - | yes | 1.4 | 3.2 | -2.3 | 8 | no | no | no |
| **14** | 10 | M | *TGFßR1* | In frame | no | - | yes | 2.3 | 3.0 | -2.0 | 2 | yes | no | no |
| **15** | 8 | M | *FBN1* | PTC | no | - | yes | 0.7 | 3.4 | -2.4 | 7 | yes | no | no |
| **16** | 10 | F | *FBN1* | Missens | yes | no | yes | 3.3 | 2.9 | -1.3 | 3 | no | no | no |
| **17** | 11 | M | *FBN1* | Missense Cys- | yes | yes | yes | 3.0 | 4.8 | 2.5 | 5 | no | no | no |
| **18** | 17 | M | *FBN1* | Missense non-Cys | no | - | yes | 3.3 | 2.9 | -3.2 | 9 | yes | no | yes |
| **19** | 8 | F | *FBN1* | PTC | no | - | yes | 2.5 | 2.7 | -2.9 | 5 | no | no | no |
| **20** | 10 | M | *FBN1* | Missense Cys+ | yes | yes | no | -0.3 | 1.9 | -2.2 | 3 | yes | no | no |
| **21** | 11 | M | *FBN1* | Missense Cys- | no | - | yes | 2.6 | 4.3 | 1.6 | 2 | yes | no | no |
| **22** | 18 | M | *FBN1* | Missense Cys- | no | - | yes | 3.7 | 1.5 | 1.6 | 5 | yes | no | no |
| **23** | 7 | F | *SMAD3* | - | no | - | yes | 0.1 | 4.1 | -0.4 | 2 | yes | no | no |
| **24** | 8 | M | *SMAD3* | PTC | no | - | no | 0.6 | 1.7 | -0.9 | 1 | no | no | no |
| **25** | 13 | F | *FBN1* | Missense Cys- | yes | yes | yes | 3.9 | 4.3 | -3.5 | 7 | no | no | no |
| **26** | 18 | M | *FBN1* | In frame del/dup | no | - | yes | 3.7 | 3.6 | -3.1 | 8 | yes | no | no |
| **27** | 16 | M | *FBN1* | Missense non-Cys | no | - | yes | 3.6 | 1.9 | 0.7 | 9 | yes | yes | no |
| **28** | 20 | M | *FBN1* | PTC | yes | no | yes | 1.4 | 4.1 | -2.5 | 9 | yes | no | no |

*preventive aorta replacement surgery

Shaded participants did not complete the training program and were excluded from the training impact analysis.

M: male; F: female; PTC: premature termination codon; Cys+: cysteine gain variant; Cys-: cysteine loss variant; Non-Cys: non-cysteine gain variant

**Supplemental Table 2.** Comparison of baseline characteristics according to systemic score and the presence or absence of scoliosis and ectopia lentis

|  | **Systemic score** | | | **Scoliosis** | | | ***Ectopia lentis*** | | |
| --- | --- | --- | --- | --- | --- | --- | --- | --- | --- |
|  | **< 7** | **≥ 7** | **P-value** | **No** | **Yes** | **P-value** | **No** | **Yes** | **P-value** |
| **Number of patients** | 14 | 14 |  | 10 | 18 |  | 15 | 13 |  |
| **Six-minute walk test** |  |  |  |  |  |  |  |  |  |
| Distance covered (z-score) | -0.7  (-2.3; -0.5) | -0.8  (-1.1; 0.0) | 0.4 | -0.6  (-0.8; 0.0) | -0.8  (-2.2; -0.4) | 0.3 | -0.8  (-2.3; -0.5) | -0.6  (-0.8; -0.4) | 0.3 |
| **Maximal exercise test** |  |  |  |  |  |  |  |  |  |
| Percent-predicted VO_2_max (%) | 70.0  (60.4; 74.6) | 54.1  (45.6; 69.9) | 0.069 | 61.9  (53.2; 68.4) | 66.5  (52.5; 74.6) | 0.5 | 61.2  (50.0; 73.0) | 66.9  (55.1; 72.2) | 0.7 |
| Impaired VO_2_ max  (< 80% of predicted VO_2_max) | 11  (78.6%) | 14  (100.0%) | 0.2 | 9  (90.0%) | 16  (88.9%) | > 0.9 | 14  (93.3%) | 11  (84.6%) | 0.6 |
| Percent-predicted VAT (%) | 38.4  (32.2; 40.5) | 29.5  (25.5; 36.8) | **0.021** | 35.3  (29.9; 40.0) | 33.8  (28.5; 38.7) | 0.6 | 32.2  (28.5; 38.2) | 38.1  (29.9; 40.0) | 0.3 |
| Impaired VAT  (< 55% of predicted VO_2_max) | 14  (100.0%) | 13  (92.9%) | > 0.9 | 10  (100.0%) | 17  (94.4%) | > 0.9 | 14  (93.3%) | 13  (100.0%) | > 0.9 |

Values were expressed as number of patients (%) or median (Q1; Q3).

VAT: ventilatory anaerobic threshold; VO_2_max: peak oxygen consumption.

**Supplemental Table 3.** Evolution of percent-predicted VAT (%) during the study

| **ID** | **M-3** | **M0** | **M3** | **M6** |
| --- | --- | --- | --- | --- |
| **1** | 29.9 | 32.6 | 41.4 | 40.0 |
| **2** | 38.7 | 44.7 | withdrawal | |
| **3** | 38.2 | 38.4 | 44.8 | 41.5 |
| **4** | 29.0 | 36.2 | 39.7 | 45.1 |
| **5** | 40.0 | 37.7 | 27.6 | withdrawal |
| **6** | 33.6 | 40.2 | 56.2 | 52.5 |
| **7** | 32.2 | 35.0 | 43.7 | withdrawal |
| **8** | 32.5 | 34.0 | 32.7 | 36.9 |
| **9** | 43.5 | 40.0 | 46.8 | 47.3 |
| **10** | 38.1 | 36.6 | 39.3 | 42.6 |
| **11** | 44.9 | 52.8 | 53.5 | 53.8 |
| **12** | 56.5 | 57.4 | 54.9 | withdrawal |
| **13** | 29.0 | 26.9 | 29.5 | 31.1 |
| **14** | 34.0 | 39.0 | missing visit | 49.1 |
| **15** | 36.8 | 31.7 | 37.1 | 42.8 |
| **16** | 40.5 | 41.0 | withdrawal | |
| **17** | 38.6 | 39.1 | 43.2 | withdrawal |
| **18** | 23.5 | 24.4 | 28.4 | 25.6 |
| **19** | 30.8 | 40.0 | 29.1 | 41.2 |
| **20** | 34.4 | 38.6 | 44.5 | 46.9 |
| **21** | 32.2 | 33.2 | 38.8 | 39.8 |
| **22** | 28.5 | 30.7 | 39.6 | 39.4 |
| **23** | 39.6 | 40.0 | 48.9 | 57.4 |
| **24** | 52.2 | 48.2 | 57.2 | 63.1 |
| **25** | 25.5 | 26.5 | withdrawal | |
| **26** | 18.4 | 17.6 | 19.3 | 25.2 |
| **27** | 21.6 | 29.7 | withdrawal | |
| **28** | 27.1 | 29.3 | 32.1 | 35.6 |
